# Assessment of the Prevalence of Diabetic Gastroparesis and Validation of Gastric Emptying Scintigraphy for Diagnosis

**DOI:** 10.4274/mirt.61587

**Published:** 2017-02-01

**Authors:** Zeinab Alipour, Foad Khatib, Seyed Masoud Tabib, Hamid Javadi, Esmail Jafari, Leila Aghaghazvini, Ali Mahmoud-Pashazadeh, Iraj Nabipour, Majid Assadi

**Affiliations:** 1 Bushehr University of Medical Sciences, Bushehr Medical University Hospital, Department of Internal Medicine, Division of Gastroenterology, Bushehr, Iran; 2 Golestan University of Medical Sciences, Golestan Research Center of Gastroenterology and Hepatology, Gorgan, Iran; 3 Bushehr University of Medical Sciences, The Persian Gulf Nuclear Medicine Research Center, Bushehr, Iran; 4 Tehran University of Medical Sciences, Shariati Hospital, Department of Radiology, Tehran, Iran; 5 Bushehr University of Medical Sciences, The Persian Gulf Tropical and Infectious Diseases Research Centre, Bushehr, Iran

**Keywords:** Gastroparesis, Gastric emptying, Diabetes, scintigraphy

## Abstract

**Objective:**

Gastroparesis is defined as delayed gastric emptying and is a common medical condition in diabetic patients. Scintigraphy is commonly used as a standard diagnostic procedure for the quantitative assessment of gastroparesis. The aims of this study were to determine an optimum imaging time for the diagnosis of gastroparesis, to assess the prevalence of gastroparesis, to evaluate the correlation between endoscopy and scintigraphy findings as well as the correlation between gastric emptying with patient genders, blood glucose concentration, and functional dyspepsia.

**Methods:**

Gastric emptying was assessed in 50 diabetic patients with a mean age of 50.16 years. For evaluation of gastric emptying, a test meal containing 2 pieces of toast, 120 cc non-labeled water and fried egg labeled with 1 mCi of 99mTc was given to each patient. The scintigraphy was performed immediately after ingestion and was repeated at 1, 1.5, 2 and 4 hours after ingestion. In some patients, an additional 90-minute dynamic scan was also acquired.

**Results:**

The prevalence of gastroparesis in this study population was determined as 64%. Also, the results of this study revealed that a 4-hour scan after ingestion is more relevant than a 90-minute dynamic scan for the evaluation of delayed gastric emptying. There was no statistically significant difference between 1-hour and 2-hour scans, 1-hour and 90-minute scans, 2-hour and 90-minute scans, 2-hour and 4-hour scans. Likewise there was no significant correlation between blood glucose levels, gender and calculated values of gastric emptying time in all groups.

**Conclusion:**

According to our findings, it can be suggested that the prevalence of gastroparesis is higher than that mentioned in some previous studies. Also, this study indicates that a gastric emptying scintigraphy at 2 and 4 hours after meal ingestion might provide the anticipated clinical information in diabetic patients with dyspepsia without other evident reasons.

## Abstract (tr)

**Amaç:**

Gastroparezi gecikmiş mide boşalması olarak tanımlanır ve diyabetik hastalarda sık rastlanan bir tıbbi durumdur. Gastroparezinin kantitatif değerlendirmesi için sintigrafi sıklıkla standart bir diagnostik yöntem olarak kullanılmaktadır. Bu çalışmanın amaçları gastroparezi tanısında optimum görüntüleme zamanını belirlemek, gastroparezi prevalansını belirlemek, endoskopi ve sintigrafi bulguları arasındaki uyumu ve mide boşalması ile cinsiyet, kan glukoz düzeyi ve fonksiyonel dispepsi arasındaki ilişkiyi incelemektir.

**Yöntem:**

Ortalama yaşları 50,16 olan 50 diyabetik hastanın mide boşalması değerlendirildi. Mide boşalmasını değerlendirmek için her hastaya 2 dilim tost, 120 cc işaretlenmemiş su ve 1 mCi 99mTc ile işaretlenmiş kızartılmış yumurtadan oluşan bir öğün verildi. Oral alımın hemen sonrasında 1., 1,5., 2. ve 4. saatlerde sintigrafi yapıldı. Bazı hastalarda ek olarak 90. dakika dinamik görüntülemesi de uygulandı.

**Bulgular:**

Çalışma grubunda gastroparezi prevalansı %64 olarak bulundu. Aynı zamanda oral alımdan sonra 4. saat görüntülemesinin gecikmiş mide boşalmasının değerlendirilmesinde 90. dakika dinamik görüntülemeden daha uygun olduğu bulundu. Birinci saat ve 2. saat, 1. saat ve 90. dakika, 2. saat ve 90. dakika, 2. saat ve 4. saat görüntülemeleri arasında istatistiksel olarak anlamlı fark yoktu. Benzer şekilde, tüm gruplarda kan glukoz düzeyi, cinsiyet ve hesaplanan mide boşalma zamanı arasında anlamlı bir ilişki saptanmadı. Görüntülemeler arasında cinsiyet, kan şekeri düzeyi ve mide boşalma süreleri açısından da fark tespit edilmedi.

**Sonuç:**

Sonuçlarımıza göre gastroparezi prevalansının daha önce bildirilenden daha yüksek olduğu öne sürülebilir. Benzer şekilde, bu çalışma gıda alımından 2 ve 4 saat sonra uygulanan mide boşalma sintigrafisinin dispeptik şikayetleri olan ancak diğer bariz nedenler bulunmayan diyabetik hastalarda gerekli klinik bilgiyi verebileceğini öngörmektedir.

## INTRODUCTION

Gastroparesis is a medical condition that is defined as delayed gastric emptying in the absence of mechanical obstruction, and is known as one of the most common side effects of diabetes mellitus (1,2). Abdominal bloating, satiety and upper abdominal pain symptoms that are associated with delayed gastric emptying in diabetic patients (3,4,5,6).

Amongst all other methods, radionuclide imaging of gastric emptying is accepted as the gold standard to evaluate patients with symptoms related to gastric emptying disorders (7). Since the first use of radionuclide scintigraphy to evaluate gastric emptying in 1966, it has become an acceptable procedure in clinical practice by enabling noninvasive quantitative survey of gastric emptying (8). In this procedure, a solid or liquid meal labeled with radionuclide is used to provide a gastric count as an index of gastric disorder (9).

Based on radionuclide scintigraphy results on gastric emptying in diabetic patients, the prevalence of delayed gastric emptying has been reported as 25-55% and 30% in patients with type 1 and 2 diabetes, respectively (10,11,12). Also, it has been determined that 29% of patients with gastroparesis had diabetes mellitus (13).

The evidence shows that several features of diabetic patients can have an effect on gastric emptying time, thus must be considered during interpretation. For example, acute alterations in blood glucose concentration can change gastric emptying time of both liquid and solid meals (14). Although the pathogenesis of gastroparesis has been attributed to poorly controlled hyperglycemia, some researches did not show a correlation between gastroparesis and autonomic dysfunction (4,12,15). Another study reported a correlation between diabetic gastroparesis and cardiovascular disease and retinopathy (16).

Recently, the Society of Nuclear Medicine and Molecular Imaging and the American Society of Neurogastroenterology and Motility agreed on application of a standard diet and standard imaging protocol for the evaluation of gastric emptying. This procedure has been standardized based on a study on 123 healthy cases, with a standard meal and several imaging times (17). While many studies have been performed on the assessment of gastric emptying by scintigraphy, some issues are still controversial.

In a research study on the diagnosis of gastroparesis, it’s mentioned that food remnants were observed within the stomach by endoscopy in patients with normal scintigraphy findings (18,19). Existence of remaining food in the stomach 12 hours after fasting represents gastroparesis in the absence of gastric outflow obstruction. However, the Diabetes Care Standards, published in 2014, did not include endoscopy as a diagnostic method for gastroparesis (18,19).

Nevertheless, it is reported that some patients require scintigraphic evaluation for gastric emptying (20,21,22,23). This may include a group of patients with rapid gastric emptying associated with nausea, bloating and satiety. Similar observations have also been reported in a group of patients with functional dyspepsia. On the other hand, rapid gastric emptying may occur at the beginning of type 2 diabetes in some patients. Many patients present with symptoms that are not distinguishable from gastroparesis. Also, it has been reported that rapid gastric emptying was more common than delayed gastric emptying in patients with autonomic dysfunction.

Further evaluation is needed to assess scintigraphy imaging at the 30- or the 60-minute and the 2- or the 4-hour scans for the diagnosis of rapid gastric emptying (8).

Therefore, the aim of the current study was to find the optimum imaging times for diagnosis of gastroparesis and rapid gastric emptying, and to assess the prevalence of gastroparesis in diabetic patients with dyspepsia. The relations between endoscopy and scintigraphy results for the diagnosis of gastroparesis were also evaluated. We also assessed the correlation between gastric emptying and gender and blood glucose concentration, as well as that between functional dyspepsia and gastric emptying disorders.

## MATERIALS AND METHODS

Fifty diabetic patients (34 men and 16 women) with an age range of 24 to 65 years (mean of 50.16±12.11 years) who have been referred to the Gastroenterology Clinic at our hospital were included in this study. Patients with normal endoscopy who did not have any exclusion criteria were referred to nuclear medicine center for evaluation of gastric emptying. The study was approved by the Bushehr University of Medical Sciences (protocol number: 2345)

Exclusion criteria in this study included heart disease (MI, heart failure and heart valve problems), metabolic disorders (hypothyroidism, kidney failure and liver failure), rheumatic diseases (lupus and scleroderma), history of peptide ulcer disease, history of surgery affecting gastric emptying (vagotomy, gastric bypass), and use of drugs affecting gastric emptying (anticholinergics, prokinetic drugs and opioids).

Before initiation of the study, all study stages were explained to the patients and consent form was obtained. They also had an option to withdraw from study participation at any time.

In case of medications that could affect gastric emptying, all patients were asked to discontinue them for 48 hours before the study, with the supervision of a physician. Patients were also asked to stop using tobacco at least at the morning of the study and were explained that they would not be allowed to smoke until the end of the study. Patients were allowed to take medications which do not affect gastric emptying as well as insulin as prescribed dosages.

In order to decrease the influence of daily activities on gastric emptying, the study was performed in the morning after at least 6 hours of fasting before the study. On the day of the study, blood glucose concentration of each patient was checked and if it was less than 275 mg/dL, the study was continued. Then each patient was given a test meal containing 2 pieces of toast, 120 cc non labeled water and fried egg labeled with 1 mCi of ^99m^Tc-sulfur colloid.

For evaluation of gastric emptying by scintigraphy, scanning was started in a static acquisition mode immediately after ingestion for each patient (time 0). Then, the study was repeated at post-ingestion times of 30 minutes in 29 patients, 60 minutes in 28 patients, 90 minutes in 27 patients, 2 hours in 48 patients, and also 4 hours in 24 patients. In addition, the study was performed with the 90-minute dynamic mode in 13 patients. The study period was also increased so as to improve the accuracy of the obtained results. Patients who had not taken the labeled meal completely or had vomited before the 2-hour scan were excluded from the study. Also, if there was no possibility of a 4-hour scan (such as vomiting), scanning data of other times was used. The scan was acquired in the anterior and posterior projections in a fixed supine position. Data was acquired with a gamma camera (Pegsys, ADAC Lab, USA) equipped by a low-energy high-resolution collimator. Region of interest of the stomach was initially drawn manually, then with the corresponding software. All measured activities were corrected using the decay factor (DF) expressed as:

DF=exp(-ln 2×t/361)

Where t refers to the time (minutes) elapsed after the first measurement.

Delayed gastric emptying or gastric retention was defined as 90% retention at the 1-hour, more than 60% at the 2-hour, and more than 10% at the 4-hour scans. Retention less than 30% at the 1-hour or less than 70% at 30-minute scans were considered as rapid gastric emptying (3,17,24). Retention more than 65% in the 90-minute scan was considered as delayed gastric emptying.

### Statistical Analysis

Continuous variables were compared with unpaired t-test and categorical variables were compared with chi-square analysis. A p-value less than 0.05 were considered as statistically significant. For statistical analysis, SPSS for Windows software package (Release 18, SPSS lnc., Chicago, llinois) was used.

## RESULTS

**Figure 1:**
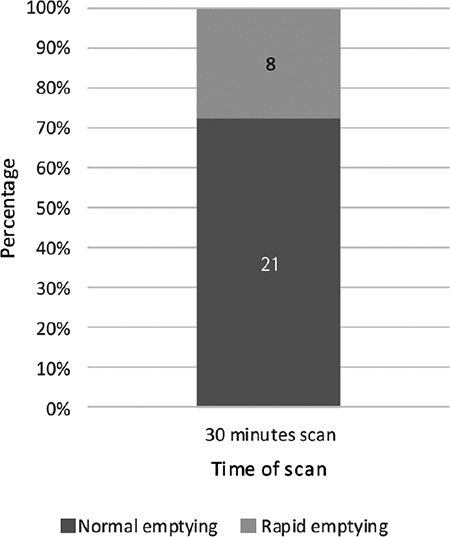
Rapid gastric emptying based on 30-minute gastric emptying scintigraphy

**Figure 2:**
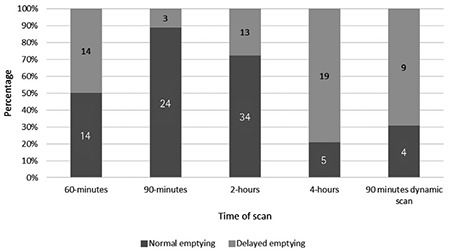
Rapid gastric emptying based on five time points of gastric emptying scintigraphy

According to the analysis of the obtained data, the mean fasting blood sugar (FBS) was determined as 165.63±50.42 mmol/L. Scintigraphy test was done at 30 minutes, 1, 1.5, 2 and 4 hours after eating radioactivity labeled meal thus creating 5 groups of patients. Within the first group of patients (29 patients), eight patients (27.6%) had rapid gastric emptying while others were normal (Table 1, Figure 1). In the second group of 28 patients, 14 patients (50%) had delayed gastric emptying and others were normal (Table 2, Figure 2). In the third group of patients (27 patients), delayed gastric emptying was observed in 3 (11.1%) with 24 (88.9%) normal results (Table 2, Figure 2). Within the fourth group in the 2-hour post ingestion evaluation, 13 (27.7%) and 34 (72.3%) patients showed delayed and normal gastric emptying, respectively (Table 2, Figure 2). Finally, within the fifth group in the 4-hour post ingestion, delayed and normal gastric emptying were observed in 19 (72.9%) and five (20.8%) patients, respectively (Table 2, Figure 2).

Among 13 patients who underwent dynamic scanning of gastric emptying, nine (69.2%) cases showed delayed gastric emptying while four (30.8%) patients were normal (Table 2, Figure 2). In none of the patients, evidence of gastroparesis was detected on endoscopy.

It was observed that patient gender did not have an effect on gastric emptying.

Based on the statistical analysis of the data, a significant correlation between FBS level and gastric emptying score was only detected at the 30-minute scan (p=0.04). Rapid gastric emptying at the 30-minute scan was associated with delayed gastric emptying at 1 hour (p value <0.001).

Because of limited number of cases who completed both tests, the correlation between the 1 and 4 hours scanning results could not be evaluated. However, the reported abnormality rate was significant in both time points (50% and 79%, respectively for 1 and 4 hours).

According to the available data, there was no statistical correlation between the 1-hour scan and the 90-minute static scan (p=0.82). Also, there was no statistically significant difference between the 2-hour scan and the 90-minute dynamic scan (p=0.109).

In comparison of the results of the 2- and 4-hour scans in 24 patients, 12 and 19 cases had delayed gastric emptying, respectively, but there was no statistically significant difference between these two groups (p=0.32).

In the evaluation of the 90-minute dynamic scan and 4-hour scan, nine cases out of 11 patients and 11 cases out of 11 patients showed delayed gastric emptying, respectively, confirming a clear advantage of the 4 hour scan in the evaluation of gastric emptying (p=0.021).

## DISCUSSION

Based on the results of the 30-minute gastric emptying scan performed on 29 patients, eight cases (27.6%) showed rapid gastric emptying. This finding was in accordance with the results of a similar study published in 2009, in which 28 cases of 129 patients (22%) showed rapid emptying of solid materials. The prevalence of rapid gastric emptying in type 2 diabetes was slightly higher in the mentioned study. This discrepancy could be related to the different procedures used in the two studies. In their study, a 1-hour scan has been used for evaluation of gastric emptying while we used a 30-minute scan (25).

It was previously shown that rapid gastric emptying occurs in both diabetic and functional dyspepsia (26), however, exactly how many patients have rapid gastric emptying is not clear because of complications of or accompaniment with diabetes. Therefore, a clinical trial is required to compare rapid gastric emptying in diabetic patients with dyspepsia and in non-diabetic patients with functional dyspepsia. Unfortunately, in most studies, the control group was selected from healthy people without symptoms of dyspepsia.

According to the statistical analysis of our data, there was a positive correlation between rapid gastric emptying in the 30-minute scan and FBS levels (p=0.048). This finding confirms the finding of a previous study showing that a higher FBS was associated with rapid gastric emptying (27).

In a previous study, C breath test-octanoic acid and T1/2 were used to assess gastric emptying (28). The prevalence of delayed gastric emptying in patients with type 1 diabetes has been reported as 33.7% in that study (28), which is different from our results. This difference could be related to the different procedures applied in the two studies.

In another comprehensive study, the prevalence of gastroparesis was reported as 24.2 in 100000, and the cumulative incidence of gastroparesis in a 10-year period was reported as 5.2% and 1% in type 1 and 2 diabetes respectively, that seems to be very low. However, patients with gastrointestinal symptoms included in that study were not assessed for gastric emptying with scintigraphy, which could have affected their findings (29,30).

In two other studies published in 1983 and 2001, it was determined that 60% of patients with long-lasting type 1 diabetes had suffered from gastrointestinal symptoms of gastroparesis. Although the method of patient selection was different from our study, their results were close to that of ours. It should be noted that these studies were conducted before the routine use of insulin for intensive treatment of type 1 diabetes (4,31,32).

In another study, delayed gastric emptying of solid materials had been discovered in 56% of patients (10). Although this amount was less than our results, it should be considered that in their study gastric emptying half time has been used for the evaluation of gastroparesis (10). Anudeep et al. (12) evaluated the prevalence and predictors of delayed gastric emptying among 140 patients with type 2 diabetes mellitus in which delayed gastric emptying was detected in about 29 % (40/140) of type 2 diabetes patients.

The higher prevalence of gastroparesis in our study can be due to the strict inclusion criteria of our study. On the other hand, gastroparesis was accepted as delayed gastric emptying in the 1-hour scan in our study while in several other studies the 2- or 4-hour scans were taken into consideration.

Gastric emptying study using a 90-minute dynamic scan is usually associated with increased costs and may lead to increased work for both the patient and the staff.

Based on the findings of the current attempt that showed the superiority of the static scan over the dynamic scan, a 2- and/or 4-hour scan instead of a 90-minute scan is recommended in diabetic patients who undergo scintigraphic studies. This is mainly because more cases have been diagnosed with delayed gastric emptying with the 4-hour scan who had initially been recognized as normal with the 90-minute dynamic scan. Therefore, based on the current study results, the most appropriate time for gastric emptying study seems to be either 2 or 4 hours both post meal ingestion to detect as much cases as possible. This could lead to a significantly better management of diabetic patients. While this is a significant advantage, it is not the only superiority of the static scan over dynamic scanning for detecting gastric emptying. Using the static scan, both the patients and the staff who are involved in the scintigraphy exam would spent less time to perform the test as compared to the 90-minute continuous scan. Therefore, it is easier for both groups, patients and staff, to perform the static scan. Additionally, a dynamic scan might exert higher workload to the single-photon emission computed tomography system in comparison to the static one, which is another positive aspect of the static scan.

Regarding the follow-up study, the finding of our survey highlighted the importance of gastric emptying study in diabetic patients. Our results showed that the prevalence of gastroparesis in diabetic patients is more than what it was originally thought. The high rate of patients with delayed gastric emptying indicates that testing should be considered to enable a tailored management of diabetic patients.

Although we tried to keep patient groups homogeneous with applying different scan times and including as much eligible patients as possible to reinforce the validity of our findings, we experienced some limitations in this regard, which is in fact the main drawback of the current survey. Future studies assessing the optimum scanning time of diabetic patients with more uniform groups and higher number of patients should be conducted both to obtain more solid data and to verify our findings.

## CONCLUSION

Based on the study findings, it can be suggested that the prevalence of gastroparesis is higher than the previously reported rates. Although a larger population is needed for overall conclusion, this study can provide context for further studies, which would have been otherwise impossible due to ethical considerations.

According to our findings, it seems that in diabetic patients suffering from dyspepsia without other evident reasons, 2 and/or 4 hours after meal ingestion is an appropriate time to conduct a gastric emptying scintigraphy. Furthermore, we conclude that the prevalence of gastroparesis is higher than the previously reported rates.

## Figures and Tables

**Table 1:**
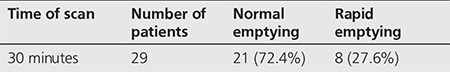
Patient data at 30 minute gastric emptying scintigraphy

**Table 2:**
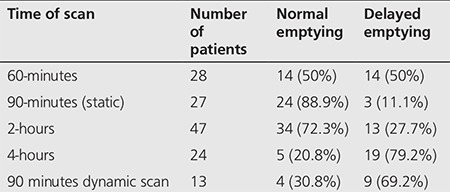
Patient data at five time points of gastric emptying scintigraphy
